# Auger Emitting [^161^Tb]Tb-PSMA-617 Radioligand Therapy in mCRPC with Peritoneal Carcinomatosis

**DOI:** 10.1007/s13139-025-00973-7

**Published:** 2025-12-02

**Authors:** Florian Rosar, Arne Blickle, Moritz B. Bastian, Tilman Speicher, Andrea Schaefer-Schuler, Caroline Burgard, Stephan Maus, Samer Ezziddin

**Affiliations:** Department of Nuclear Medicine, Saarland University – Medical Center, Kirrberger Str. 100, Geb. 50, 66421 Homburg, Germany

A 72-year-old patient with metastatic castration-resistant prostate cancer (mCRPC, diagnosis of mCRPC 9 years ago, initial diagnosis of prostate cancer 13 years ago) with diffuse peritoneal, multiple lymph node, and soft tissue metastases received prostate-specific membrane antigen (PSMA) targeted radioligand therapy (RLT) with [^161^Tb]Tb-PSMA-617. The patient had previously undergone multiple lines of therapy, including androgen deprivation therapy (ADT), androgen receptor signaling inhibitors (ARSI) with enzalutamide and abiraterone, taxane-based chemotherapy with docetaxel, and RLT with [^177^Lu]Lu-PSMA-617 (17 cycles across 3 series with a cumulative activity of 118.8 GBq). We opted for [^161^Tb]Tb-PSMA-617 therapy, as the patient, who had initially shown a favorable response to [^177^Lu]Lu-PSMA-617 over initial series and 2 rechallenge series, eventually presented re-progression with diffuse peritoneal metastases. The patient was treated with four cycles of [^161^Tb]Tb-PSMA-617 with a mean administered activity of 5.1 ± 0.3 GBq per cycle (range: 4.7–5.5 GBq), totaling a cumulative administered activity of 20.5 GBq. Following treatment, PSMA PET/CT imaging revealed a remarkable regression of peritoneal, nodal, and soft tissue metastases. The presented figure shows baseline and post therapeutic [^68^Ga]Ga-PSMA-11 PET/CT after 4 cycles of [^161^Tb]Tb-PSMA-617 RLT. Pre-therapeutic PSMA PET/CT imaging revealed diffuse peritoneal, multiple lymph node, and soft-tissue metastases, whereas post-therapeutic PSMA PET/CT imaging demonstrated marked regression of these lesions (Fig. 1). In line with the imaging findings, the patient demonstrated a biochemical partial remission, with PSA declining from a baseline of 312 ng/mL to 39 ng/mL, representing an 87.5% decrease. Prior to initiation of [^161^Tb]Tb-PSMA-617 RLT, the most severe adverse events observed were grade 2 anemia, grade 2 renal impairment, and grade 2 xerostomia, following CTCAE criteria. No worsening or newly appearing adverse events were recorded following [^161^Tb]Tb-PSMA-617 RLT.

Terbium-161 is an emerging radionuclide that is attracting growing interest for its potential use in RLT [1]. To date, terbium-161 has demonstrated promising results in preclinical studies, preliminary clinical investigation, and most recently in a first prospective phase I/II clinical trial [2–4]. The radionuclide terbium-161 exhibits physical decay characteristics closely resembling those of lutetium-177, including comparable β⁻ emission energies (Lutetium-177: 133 keV vs. Terbium-161: 154 keV) and similar half-lives (Lutetium-177: 6.647 days vs. Terbium-161: 6.906 days). A key distinguishing feature of terbium-161 is its emission of a significantly higher number of low-energy conversion electrons and Auger electrons. These electrons possess an ultra-short tissue penetration range, resulting in a relatively high linear energy transfer. This translates into higher local dose deposition by terbium-161 in comparison to lutetium-177 [5].

This property may be especially advantageous in targeting micrometastatic disease, such as that observed in cases of diffuse peritoneal carcinomatosis. To the best of our knowledge, this case represents the first demonstration of the effectiveness of [^161^Tb]Tb-PSMA-617 in the treatment of peritoneal carcinomatosis. Current literature only encompasses a limited number of studies exploring the potential of [^161^Tb]Tb-PSMA-617 RLT mandating further explorative studies to substantiate its evidence, e.g. in the setting of peritoneal carcinomatosis. This case encourages the consideration of terbium-161 for use in PSMA RLT, especially in clinical scenarios where control of diffuse or microscopic tumor spread is critical.

**Fig. 1:**
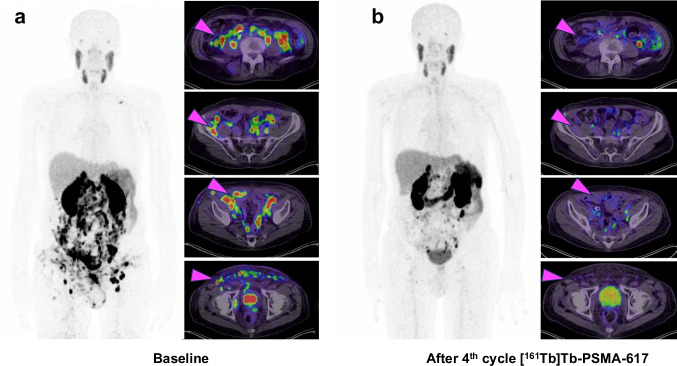
A 72-year-old mCRPC patient with diffuse peritoneal, multiple lymph node, and soft tissue metastases (exemplary magenta arrows) received PSMA RLT with [^161^Tb]Tb-PSMA-617. **a**) depicts a [^68^Ga]Ga-PSMA-11 PET/CT scan pre PSMA RLT and **b**) depicts a [^68^Ga]Ga-PSMA-11 PET/CT scan after four cycles of [^161^Tb]Tb-PSMA-617 RLT with regression of metastases

## Data Availability

The corresponding data are not publicly available; however, can be provided by the corresponding author upon reasonable request.

## Declarations

### Clinical Trial Number

NCT04833517, Registry: Prospective REgistry of Targeted RadionucLide TherapY in Patients With mCRPC (REALITY Study), registration date: 2016–01-01.
